# Disparate access to breast cancer screening and treatment

**DOI:** 10.1186/s12905-022-01793-z

**Published:** 2022-06-22

**Authors:** Maria Castaldi, Abbas Smiley, Katharine Kechejian, Jonathan Butler, Rifat Latifi

**Affiliations:** 1 New York Medical College, Valhalla, NY 10595 USA; 2 Department of Surgery, Westchester Medical Center, Valhalla, NY 10595 USA; 3 Department of Surgery, NYC Health + Hospitals/Jacobi, Bronx, NY 10461 USA

**Keywords:** Breast cancer screening, Barriers, Access, Patient navigation

## Abstract

**Background:**

Barriers to breast cancer screening remain despite Medicaid expansion for preventive screening tests and implementation of patient navigation programs under the Affordable Care Act. Women from underserved communities experience disproportionately low rates of screening mammography. This study compares barriers to breast cancer screening among women at an inner-city safety-net center (City) and those at a suburban county medical center (County). Inner city and suburban county medical centers’ initiatives were studied to compare outcomes of breast cancer screening and factors that influence access to care.

**Methods:**

Women 40 years of age or older delinquent in breast cancer screening were offered patient navigation services between October 2014 and September 2019. Four different screening time-to-event intervals were investigated: time from patient navigation acceptance to screening mammography, to diagnostic mammography, to biopsy, and overall screening completion time. Barriers to complete breast cancer screening between the two centers were compared.

**Results:**

Women from lowest income quartiles took significantly longer to complete breast cancer screening when compared to women from higher income quartiles when a barrier was present, regardless of barrier type and center. Transportation was a major barrier to screening mammography completion, while fear was the major barrier to abnormal screening work up.

**Conclusion:**

Disparity in breast cancer screening and management persists despite implementation of a patient navigation program. In the presence of a barrier, women from the lowest income quartiles have prolonged breast cancer screening completion time regardless of center or barrier type. Women who experience fear have longest screening time completion. Future directions aim to increase resource allocation to ameliorate wait times in overburdened safety-net hospitals as well as advanced training for patient navigators to alleviate women’s fears.

## Introduction

Under the Affordable Care Act, Medicaid expansion allowed insurance coverage for screening mammography. Expanded eligibility by acquiring insurance improves access to preventative care [1]. The increase in Medicaid coverage allowed improved access to screening mammography in women from low-income groups. Communities that would otherwise have difficulty obtaining screening studies are more likely to take advantage of expanded opportunities via state-funded programs and have improved access to breast health and screening mammography [2].

Governor Andrew M. Cuomo launched ‘Get Screened, No Excuses’ campaign to increase mammographic screening rates in New York [3]. This initiative has been one of the nation’s most aggressive efforts to improve access to breast cancer screening via Patient Navigation Program (PNP) development. Patient navigation (PN) describes interventions involving people or organizations that aim to promote access to healthcare for communities that have been historically marginalized and vulnerable [4]. Patient navigation services have been reported to have significant impact on adherence to breast cancer treatment and serve as an intervention tool to aid in breast cancer screening [5]. Patient navigators (PNs) offer support and assistance for follow through with screening and associated diagnostics and treatment as needed.

Analysis of breast centers’ initiatives and outcomes on breast cancer screening rates supported by the New York State Patient Navigation Program for Breast Cancer Screening showed that age, screening stage, and income were the most important variables associated with compliant breast cancer screening [2]. PN captured women delinquent in breast cancer screening and increased screening by 800 women per year. However, despite implementation of the Governor’s Patient Navigation Program, disparate screening rates with prolonged timliness to care persisted in those with low socioeconomic status (SES) treated at a safety-net hospital. Further, despite navigation implementation in safety net-hospitals, barriers were not overcome to improve timeliness to screening. Poorer outcomes and mortality will continue to rise due to disparate treatment rates. The authors sought to investigate barriers that could be responsible for disparate timely completion of breast cancer screening in women of lower socioeconomic status. This investigation would serve to better understand how to improve healthcare delivery and preventative measures to patients cared for in centers serving lower income communities.

Groups that have been historically marginalized are least likely to be screened for many reasons. Several studies have shown that certain categories of people, particularly the most vulnerable, due to economic and social marginalization, are excluded from health systems and require more support to overcome the multitude of barriers they encounter in accessing care [6]. Through the provisions of the Governor’s New York State Department of Health Grant, centers were awarded funding to support activities related to the grant’s breast cancer initiatives in providing screening patient navigation to women in need of breast cancer screening. Two centers identified similar target populations to increase breast cancer screening. The Affordable Care Act (ACA) signed into law in 2010, mandated insurance coverage for women in their forties. In 2015, the American College of Radiology and Society of Breast Imaging reaffirmed the benefit to yearly mammography beginning at age forty. With this, the American Society of Breast Surgeons and the American Cancer Society endorsed recommendations that average risk women undergo yearly screening mammography beginning at age forty. The breast program leaders developed similar pathways for grant project delivery; one center is a safety-net hospital (City) and the second, a county medical center (County).

## Methods

### Data source

Institutional data was collected from two breast centers accredited by the American College of Surgeons’ National Accreditation Program for Breast Centers (NAPBC) in receipt of funding from the governor’s grant for the New York State Patient Navigation Program for Breast Cancer Screening. We compare an inner-city safety net hospital (City), serving mainly those communities that have been socioeconomically marginalized and underrepresented and a second center (County), a regional suburban county hospital, serving communities with less socioeconomic disadvantage and those with healthcare insurance. Women 40 years of age or older delinquent in breast cancer screening one year or greater were eligible for enrollment for breast screening and PN services.

A PN followed women who accepted PN services for breast cancer screening through the governor’s grant at both centers via a tracking tool. Women accepted PN services at the initial encounter between patient and navigator, in person or by telephone. In person encounters occurred at breast screening and outreach events. The tracking tool included demographic and contact information, as well as barriers reported or encountered that interfered with ability to obtain a mammogram. The PN recorded patient self-reported barriers to screening on the tracking tool.

Process measures monitored included women contacted within City and County centers and from the priority communities with the offer of PN services, women referred for screening mammography, women in need of screening, total screened, positive findings and women requiring breast biopsy. The NYS DOH/Health Research Inc. [7] staff monitored the progress of City and County contractor’s progress towards work plan goals, objectives, and deliverables to meet funding criteria. Project progress and screening results were generated monthly, quarterly, semiannually, and reported to DOH.

Target and priority populations for community outreach activity were identified by collaborating with off- site community centers and local health systems, DSRIP Program Performing Provider Systems, Federally Qualified Health Centers (FQHCs), and health plans. Hospital radiology logs of missed appointments also identified those in need of breast screening.

PNs are salaried employees of both  City and County centers and receive neither financial incentives nor compensation for their work or numbers of women enrolled. PNs were selected based on prior healthcare experience, through survivorship or nursing training. City and County PNs had prior cancer navigation experience.

PNs contacted patients referred by community partners and from lists generated by affiliated health systems and radiology logs to determine need for and overdue breast cancer screening. PNs identified women in need of breast cancer screening if age ≥ 40 and not screened yearly. Upon acceptance of PN services, the PNs assessed barriers to screening, scheduled women for cancer screening services, followed women forscreening completion and connection hand off to the NAPBC navigator for further work up for abnormal mammography. Barrier assessment tools were created for each institution. The self-reported barriers to care and timely screening were grouped into child care; paid time off (PTO); transportation; fear, which included fear of procedure and fear of results; support, which included family, cultural, religious, companion to accompany; scheduling, which included assistance with scheduling appointment, missed appointment, and scheduling conflicts; and other barriers, which included all remaining barriers such as immigration, insurance, and language barriers. The PNs were a source of continual support for patients acting as liaison between patient and healthcare provider and center. The governor’s grant PN activity was integrated into the center's current education and outreach activity workflow requirement to maintain center compliance with American College of Surgeon’s NAPBC accreditation.

### Patient selection

Women 40 years of age or older, delinquent in breast cancer screening one year or greater were offered PN services. The Affordable Care Act (ACA) signed into law in 2010, mandated insurance coverage for women in their forties. In 2015, the American College of Radiology and Society of Breast Imaging reaffirmed the benefit to yearly mammography beginning at age 40. With this, the American Society of Breast Surgeons and the American Cancer Society endorsed recommendations that average risk women undergo yearly screening mammography beginning at age 40. Those who accepted and completed breast cancer screening at one of two, City or County, NAPBC-accredited breast centers between October 2014 and September 2019 were tracked. Primary outcomes of interest were identification of barriers to screening yearly breast screening.

### Statistical analyses

Comparative analysis on women aged 40 years and over in need of screening mammography who accepted PN services at City and County NAPBC-accredited breast centers was conducted. (Table 1) Frequency distribution of different barriers was compared between those who completed screening and those who did not, using chi-square test. Similarly, the frequency distribution of different barriers was compared between the two centers using chi-square test. Differences in breast cancer screening times were compared between the two centers for each barrier using t test. Additionally, differences in breast cancer screening times were compared among various barriers within each center using ANOVA. Women were classified based on income quartiles. Screening times were compared among various barriers within each income quartile using ANOVA. The prevalence of barriers within each stage of screening were compared using chi-square test. An alpha level of 0.05 was used to determine if the groups were statistically different. Data was analyzed using SPSS, version 26 (IBM Corporation, SPSS, Chicago, IL). This study was approved by the New York Medical College Institutional Review Board.

**Table 1:** Screening center elements

	City center	County center	*p* Value
Observation, N (%)	1,167 (47)	1,338 (53)	
Age (Years), mean (SD)	54.9 (9.7)	57.5 (10.3)	** < 0.001**
Median income for zip code, dollars, mean (SD)	42,899 (16,249)	71,513 (34,135)	** < 0.001**
*Screening mammography times (Days), mean (SD)*			
Navigation to screening	47.4 (74.7)	14.1 (31.7)	** < 0.001**
Screening to diagnostic	40.4 (50.0)	32.2 (58.6)	0.280
Screening to biopsy	21.0 (–)	21.2 (9.3)	0.980
Diagnostic to biopsy	52.1 (54.6)	22.5 (19.0)	**0.010**
BIRADS 4 to biopsy	51.0 (53.9)	21.8 (14.3)	**0.007**
Navigation to screening completion	53.7 (77.5)	163.7 (36.4)	** < 0.001**
Navigation to last contact	52.7 (77.1)	16.4 (36.2)	** < 0.001**
*Median income for zip code quartile, N (%)*			
Lowest quartile (0–25)	620 (53)	16 (1)	** < 0.001**
Quartile 2 (26–50)	55 (5)	570 (43)	
Quartile 3 (51–75)	461 (40)	152 (11)	
Highest quartile (76–100)	31 (3)	600 (45)	
*Barrier to screening, N (%)*			
Child care	190 (16)	99 (7)	** < 0.001**
No paid time off	287 (25)	171 (13)	** < 0.001**
Transportation	374 (32)	173 (13)	** < 0.001**
Fear	149 (13)	3 (0.2)	** < 0.001**
Support network	4 (0.3)	21 (2)	**0.020**
Scheduling issues	14 (1)	0 (0)	** < 0.001**
Other*	22 (2)	7 (0.5)	**0.001**

Bolded *p* values indicate statistical significance

*Immigration, insurance, language barriers

## Results

The sample contained 2,505 women aged 40 years or older who accepted PN services between October 2014 and September 2019. Mean (SD) age of patients was 56.2 (10) years. City included 38% of total sample size and County  62%.

### Barriers to breast cancer screening

Of the 2505 women included in the study, 39.6% identified no barrier to breast screening. The most common barriers were transportation (21.8%), no PTO (18.3%), and childcare (11.5%). Results are presented in Table 2.

**Table 2:** Top barriers to breast cancer screening

Barrier	N	Percent
No barrier	991	39.6
Child care	289	11.5
No PTO	458	18.3
Transportation	547	21.8
Fear	152	6.1
Support	25	1.0
Schedule	14	0.6
Other*	29	1.2

*Immigration, insurance, language barriers

### Barrier presence and completion of screening

Of the women who identified transportation as a barrier, 27.9% did not complete the screening, indicating that transportation was the most common barrier to completion of breast cancer screening among City and County women, regardless of income quartile. (Table 3).

**Table 3:** Barrier and completion of screening, all patients

Barrier	Not screened, N (%)	Completed screening, N (%)	*p* Value
No barrier	52 (17.5%)	939 (42.5)	** < 0.001**
Child care	48 (16.2%)	241 (10.9)	**0.008**
No PTO	68 (22.9%)	390 (17.7%)	**0.029**
Transportation	83 (27.9%)	464 (21.0%)	**0.007**
Fear	30 (10.1%)	122 (5.5%)	**0.002**
Support	4 (1.3%)	21 (1.0%)	0.528
Schedule	3 (1.0%)	11 (0.5%)	0.226
Other*	9 (3.0%)	20 (0.9%)	**0.001**

Bolded *p* values indicate statistical significance

*Immigration, insurance, language barriers

### Screening completion by type of barrier and center visited

There was a significant difference between the percentage of women in City and County who experienced no barrier and completed the breast cancer screening (Table 4). City women identified no PTO and transportation as the most common barriers to screening completion when compared to County women (Table 4). Thus, more than 58% of City women experienced no PTO and transportation as barriers to completion of screening, compared to approximately 25% of County women who faced the same barriers. More City women experienced childcare as a barrier than County women (Table 4). There was no significant difference between the percentage of women at City and County who did not complete breast cancer screening based on barrier type.(Table 4).

**Table 4:** Screening completion by barrier type and center

Barrier	Completed screening, N (%)			Did not complete screening, N (%)		
	City	County	*p* Value	City	County	*p* Value
No barrier	83 (9.2%)	856 (65.4%)	** < 0.001**	44 (16.5%)	8 (26.7%)	0.164
Child care	146 (16.2%)	95 (7.3%)	** < 0.001**	44 (16.5%)	4 (13.3%)	0.798
No PTO	228 (25.3%)	162 (12.4%)	** < 0.001**	59 (22.1%)	9 (30.0%)	0.329
Transportation	298 (33.1%)	166 (12.7%)	** < 0.001**	76 (28.5%)	7 (23.3%)	0.553
Fear	119 (13.2%)	3 (0.2%)	** < 0.001**	30 (11.2%)	0 (0%)	0.055
Support	2 (0.2%)	19 (1.5%)	**0.003**	2 (0.7%)	2 (6.7%)	0.052
Schedule	11 (1.2%)	0 (0%)	** < 0.001**	3 (1.1%)	0 (0%)	0.999
Other*	13 (1.4%)	7 (0.5%)	**0.027**	9 (3.4%)	0 (0%)	0.606

Bolded *p* values indicate statistical significance

*Immigration, insurance, language barriers

### Average screening time completion by barrier type and center visited

ANOVA test compared average breast cancer screening time completion by barrier type within City and County (Table 5). City women who experienced fear as a barrier had the longest screening time completion (41.92 days). Women who experienced no PTO as a barrier had the shortest screening time completion (25.59 days). Significant difference was observed in prevalence of different barriers among women in City (Table 5). County women who noted childcare as a barrier had the longest screening time completion (20.57 days), and those with other barriers had the shortest screening time completion (5.00 days). There was no significant difference in terms of prevalence of different barriers among County women (Table 5). T-test compared average breast cancer screening time completion by barrier type between the two centers (Table 5). Only two barriers, namely no PTO and transportation, led to significantly longer completion times in City compared to County across all income quartiles (25.69 days vs. 15.76 days, 28.77 days vs. 13.66 days, respectively).

**Table 5:** Average screening time completion by barrier type and center

Barrier	City center			County center			*p* Value**
	N	Mean (SD)	*p* Value*	N	Mean (SD)	*p* Value*	
No barrier	83 (9.2%)	31.85 (61.77)	**0.041**	856 (65.4%)	17.37 (39.43)	0.641	**0.050**
Child care	146 (16.2%)	32.33 (54.63)		95 (7.3%)	20.57 (41.52)		0.060
No PTO	228 (25.3%)	25.69 (46.67)		162 (12.4%)	15.76 (28.69)		**0.010**
Transportation	298 (33.1%)	28.77 (51.08)		166 (12.7%)	13.66 (28.08)		** < 0.001**
Fear	119 (13.2%)	41.92 (63.99)		3 (0.2%)	6.00 (5.29)		0.132
Support	2 (0.2%)	126.50 (74.25)		19 (1.5%)	9.58 (15.29)		0.267
Schedule	11 (1.2%)	41.27 (37.36)		0 (0%)			
Other*	13 (1.4%)	31.08 (27.35)		7 (0.5%)	5.00 (1.73)		**0.005**

Bolded *p* values indicate statistical significance

*Immigration, insurance, language barriers

### Average screening completion time by barrier type and income quartile

ANOVA compared average screening completion times by barrier type within each income quartile (Table 6). Within income quartile 1, there was no difference in mean screening completion time based on barrier typre. Within income quartile 2, there was a significant difference in mean screening completion time based on barrier type. The longest screening completion time was associated with childcare (56.91 days) and transportation (20.33 days). Within income quartile 3, there was no difference in mean screening completion time based on barrier type (Table 6). Within income quartile 4, there was a significant difference in mean screening completion time based on barrier type. The longest screening completion time was associated with no barrier (28.75 days) and childcare (20.19 days).

**Table 6:** Average screening completion time by barrier type and income quartile

Barrier	Income quartile 1			Income quartile 2			Income quartile 3			Income quartile 4			*p* Value
	N	Mean (SD)	*p*	N	Mean (SD)	*p*	N	Mean (SD)	*p*	N	Mean (SD)	*p*	
No barrier	50	30.24 (51.80)	0.1	516	10.08 (33.76)	**0.001**	77	33.14 (57.75)	0.07	263	28.75 (44.87)	**0.002**	** < 0.001**
Child care	81	43.69 (67.34)		11	56.91 (92.78)		87	14.45 (22.25)		62	20.19 (32.05)		** < 0.001**
No PTO	116	29.16 (47.53)		24	14.29 (20.97)		126	21.47 (44.68)		124	15.96 (29.22)		0.06
Transportation	165	27.62 (46.181)		27	20.33 (33.14)		143	26.55 (24.40)		129	15.03 (30.29)		0.07
Fear	69	46.06 (65.93)		4	17.75 (23.27)		43	38.35 (64.07)		4	6.25 (4.03)		0.52
Support							9	40.89 (59.08)		12	5.58 (3.32)		0.05
Schedule	3	30.67 (29.57)		1	3.00 (–)		7	51.29 (40.22)					0.45
Other*	7	30.86 (32.07)					7	27.43 (23.97)		6	5.17 (1.84)		0.14

Bolded *p* values indicate statistical significance

*Immigration, insurance, language barriers

### Barrier presence and breast cancer screening stage

Major differences in the prevalence of a barrier was found within each stage of screening (Table 7). Transportation and no PTO were the major barriers to completing screening mammography (27.9% and 22.9% respectively). Fear posed a barrier to screening mammography (10.1%). Of the patients who completed screening mammography, 21.0% still experienced transportation as a barrier. On the other hand, a higher percentage of women did not complete diagnostic mammography when fear was present (23.5%). Fear was the barrier that played a major role in determining who completed diagnostic mammography (Table 7). For those women requiring diagnostic mammograms, the only difference between those who completed and those who did not complete this stage was presence of fear as a barrier. One quarter of patients did not complete diagnostic mammography based on fear alone.

**Table 7:** Barrier presence and breast screening stage

Barrier	Completed screening mammography		*p* Value	Completed diagnostic mammography		*p* Value
	No, N (%)	Yes, N (%)		No, N (%)	Yes, N (%)	
No barrier	52 (17.5%)	939 (42.5)	** < 0.001**	6 (17.6%)	46 (24.7%)	0.371
Child care	48 (16.2%)	241 (10.9)	**0.008**	6 (17.6%)	31 (16.7%)	0.888
No PTO	68 (22.9%)	390 (17.7%)	**0.029**	5 (14.7%)	36 (19.4%)	0.522
Transportation	83 (27.9%)	464 (21.0%)	**0.007**	9 (26.5%)	44 (23.7%)	0.724
Fear	30 (10.1%)	122 (5.5%)	**0.002**	8 (23.5%)	20 (10.8%)	**0.040**
Support	4 (1.3%)	21 (1.0%)	0.528	0 (0%)	2 (1.1%)	0.999
Schedule	3 (1.0%)	11 (0.5%)	0.226	0 (0%)	1 (0.5%)	0.999
Other*	9 (3.0%)	20 (0.9%)	**0.001**	0 (0%)	6 (3.2%)	0.594

Bolded *p* values indicate statistical significance

*Immigration, insurance, language barriers

Top barriers interfering with timely completion of screening mammography, are transportation, 25%; PTO, 25%; and fear. Twenty-one percent of those who completed screening mammography reported transportation as the main barrier compared to 28% of those who did not complete screening. In other words, one fifth of the population who actually completed the study also experienced transportation as a barrier.

## Discussion

Our study finds women in low income quartiles experiencing longer completion times for each breast cancer screening stage as well as longer overall completion times compared to women in higher income quartiles. Among City women, fear was the most prominent and prohibitive barrier to screening mammography. However, transportation and no paid time off were the most significant barriers to timely breast cancer screening in both City and County women.

### Patient demographics

In our cohort of 2505 women, City center included 38% and County center 62% of the total sample size. This difference in sample size may be due to several factors, including a higher number of women of low SES in City, limited access to health care overall, and busy schedules with competing priorities among City women. The majority of City women fell into income quartile 1 or low SES, while the majority of County women were of high SES in income quartile 4. Women of low SES with loss of wages for medical care visits, further deter from pursuing breast cancer screening. Women with poor understanding of screening or fear of screening procedure or results, treatment facilities with lower number of mammography facilities per female population, inadequate mammography capacity, staff shortages, or limited availability of evening and weekend hours to accommodate busy work schedules are characteristic of low income communities. [8–10]. Such systemic barriers have been associated with a lower likelihood of breast cancer screenings and longer wait times for screening appointments [9, 10]. Also, patients typically struggle to keep appointments when they live far from the nearest screening facility or lack transportation [10, 11]. Thus, each of these factors may have contributed to the sample size differential between City and County.

Our main finding in comparison of City and County shows that age, screening stage, and income were the most important variables associated with timely breast cancer screening. County women were significantly older in higher income quartiles compared with women from City. Older age is a significant factor in treatment delay, which suggests that an increased number of comorbidities may be associated with longer wait times [12, 13]. In contrast, County women, although older, had faster screening times compared to younger City women. This suggests other factors at play influencing breast cancer screening outcomes in City. 

Women from the lowest SES quartiles took significantly longer to complete each screening stage as well as experiencing longest total completion times compared to women in highest quartile when a barrier was identified. Regardless of barrier type, women from the highest income quartile completed screening faster than women from the lowest income quartile. This outcome is supported by literature indicating women of low SES continue to face disparate access and treatment beyond the barriers that they face. This could be due to fewer resources to overcome barriers as well as overburdened centers serving poorer communities [9–11, 14].

### Site differences

We compared an inner-city safety-net hospital serving mainly socioeconomically disadvantaged, underinsured, and uninsured communities (City), and a regional suburban county hospital, serving insured communities with higher SES (County). City and County centers were chosen because they identified a similar population of focus, who are in greatest need of barrier reduction support provided by the Governor’s grant PNP.

There was an inherent difference in ability and capacity of the safety-net City center to provide comparative quality health care, even with the use of PNs to assist with access to screening. When comparing timeliness of screening completion between both centers, County completed the screening process in almost half the time as City. The shortest times to screening completions in the City were still greater than the longest time interval in County. Often, safety-net hospitals have fewer resources and are more financially burdened than non-safety-net hospitals, due to caring for a higher percentage of Medicaid and uninsured patients [9, 15–17]. Additionally, safety-net hospitals are experiencing an increase in caseload and wait times, as well as a shortage of provider and support staff which can slow the delivery of care to patients [14, 18]. This may add to patient anxiety, gaps in care, and perhaps worse survival outcomes due to delayed care [12, 14, 19]. Our study supports the fact that timeliness to screening is influenced by the aforementioned system barriers.

### Barriers to breast cancer screening

A Patient Navigator Tracker Spreadsheet was used to record patient demographics and self-reported barriers to screening. Patients self-reported any of the following barriers on child care, paid time off (PTO), transportation, fear, support, scheduling, as well as other via free text. Obviously, there could be some few barriers that were hidden or not recognized.

The most common barriers identified were equal across the four income quartiles: transportation, childcare, and no PTO. This supports similar findings  in the literature that show transportation to be the most common barrier overall [20, 21]. Groups that have been historically vulnerable, most commonly experience barriers such as fear of cost, fear of mammogram-associated pain and fear of receiving bad news [22, 23]. Despite free services and PN, underserved women continue to report experiential and psychological obstacles to mammography. This suggests the need for improved targeted education and outreach in these communities [22], as well as further education and training of PNs.

The majority of City women experienced no PTO and transportation as main barriers to completion of screening, compared to only one fourth of County women who faced the same barriers. City women also experienced childcare as a barrier more often than County women. Place of residence may play a role in access to health services, reliable transportation, the financial ability to take time off from work, and timely preventive health. Ultimately, these barriers delay breast cancer screening and overall screening completion and workup, which in turn, influence breast cancer treatment and mortality. The high concentration of vulnerable populations and poor areas in a City may explain why urban women are more likely to present with late-stage disease compared to those who live in suburban and rural areas [24, 25]. When comparing characteristics of urban and suburban communities, urban areas are more racially and ethnically diverse, as nonwhites make up the majority of the population, while suburban and rural areas are predominantly white [24]. Among minority women, the most common barriers to breast screening reported in the literature and supported by our study are lack of health insurance, poor knowledge about breast cancer screening, lack of physician recommendation, lack of trust in hospitals and doctors, language barriers, fear of procedure, and lack of transportation [20, 26, 27]. In addition, socioeconomically deprived communities with high unemployment rates and crime forces day-to-day survival, leaving less attention to preventative care. Lower cancer screening rates or advanced stages of breast cancer at diagnosis occur in economically and socially marginalized populations for the reasons described above [8]. Perhaps an effective way to raise awareness for breast cancer screening and prevention might be through education programs that incorporate all aspects of women's health. Incorporating breast screening while providing education for example on breastfeeding may utilize a captive audience receptive to all aspects of breast health education  [28].

Fear poses a major barrier to breast cancer screening completion. City women experiencing fear had the longest screening completion times. The only difference between women who completed the required additional diagnostic images for an abnormal screening mammogram and those who did not was the presence of fear as a barrier [29]. This finding highlights the importance of addressing not only  education but cultural attitudes and belief systems surrounding the health care system. Psychosocial factors such as fear and anxiety, fatalistic attitudes, perceived risk, misunderstanding, competing demands of caring for others, and social norms may delay diagnostic assessment and treatment [9, 26, 27]. Greater ethnic and racial diversity among City women compared to County women may account for varied cultural beliefs and a heightened sense of fear surrounding breast cancer screening, particularly as one approaches breast biopsy.

There are several limitations of this study. Firstly, structural barriers were present. Patients who were unable to make timely appointments due to overbooked mammogram schedules and lack of staff to support screening off hours such as weekends and nights were not tracked. Second, factors influencing physician recommendations were not controlled for in this study. Data on physician’s recommendations was not gathered  in order to determine if in fact some patients were following USTPS task force guidelines of screening every other year.

Future directions look to further educate those that provide patient navigation. It is well known that PNPs enhance system throughput for breast cancer treatment [5]. However, what is less known are the effects of navigation training and education levels of navigators and effects on care enhancement [5]. Patient fear toward the screening procedure and the uncertainties about the results of screening seem to be the strongest influence in decisions to pursue screening. Heath care providers and PNs must gain a better understanding of fear from the patient perspective in order to address this common barrier. Contribution of social support to influence mammography behavior has been studied. Social support can offer help directly by delivering encouragement to overcome fears and provide information and knowledge on importance of screening. It has been shown that increased performance of breast cancer screening behaviors is correlated with high levels of social support^30^. Social support through social relationships and interpersonal exchanges from family members, friends, and significant others has direct and indirect effects on health. Additionally, motivation to overcome fear relies on how passionately physicians and PNs advocate for breast screening. Both physicians and PNs while expressing the importance of screening should understand that patients may need to reach a level of comfort before they agree to be screened. Education in areas such as cultural competency and social networks may aid in alleviating fear of screening.

## Conclusion

Despite implementation of a patient navigation program, factors remain and contribute to disparate access and treatment in breast cancer screening. The mere presence of a barrier to obtain breast cancer screening revealed that women from the lowest income quartiles have prolonged screening times regardless of center or barrier type. Our study underscores the need for continued efforts on mitigating barriers and healthcare system challenges that minority and low-income women face in breast cancer care. In particular, efforts are needed to increase social support and education for women who experience fear as an obstacle to breast cancer screening completion, in addition to a financial boost for patient transportation and childcare services. It is essential to strive for improvements in health care navigation and delivery for vulnerable populations in order to improve access and ultimately better outcomes in cancer care. Further, resource allocation to overburdened safety-net hospitals remains paramount for timely care and preventative screening. Healthcare practitioners and policy makers must be aware of the aforementioned intrinsic differences between City and County populations in order to address disparate care.

## Data Availability

The datasets used and analyzed during the current study are available from the corresponding author on reasonable request.
